# Exploring the prognostic analysis of autophagy and tumor microenvironment based on monocyte cells in lung cancer

**DOI:** 10.18632/aging.205973

**Published:** 2024-06-27

**Authors:** Bo Tao, Ziming Wang, Dacheng Xie, Hongxue Cui, Bin Zhao, Juanjuan Li, Liang Guo

**Affiliations:** 1 Department of Thoracic Surgery, Shanghai Pulmonary Hospital, Tongji University School of Medicine, Shanghai, 200433, China; 2 Department of Medical Oncology, Shanghai Pulmonary Hospital and Thoracic Cancer Institute, Tongji University School of Medicine, Shanghai, 200433, China; 3 Department of Thoracic Surgery, Affiliated Hospital of Weifang Medical University, Weifang, Shandong 261031, China; 4 Department of Pulmonary Nodule Center, Shandong Public Health Clinical Center, Jinan, Shandong 250100, China

**Keywords:** The Cancer Genome Atlas, autophagy-related genes, single-cell analysis, prognostic model, immune checkpoints

## Abstract

A deep understanding of the biological mechanisms of lung cancer offers more precise treatment options for patients. In our study, we integrated data from the Gene Expression Omnibus (GEO) and The Cancer Genome Atlas (TCGA) to investigate lung adenocarcinoma. Analyzing 538 lung cancer samples and 31 normal samples, we focused on 3076 autophagy-related genes. Using Seurat, dplyr, tidyverse, and ggplot2, we conducted single-cell data analysis, assessing the quality and performing Principal Component Analysis (PCA) and t-SNE analyses. Differential analysis of TCGA data using the “Limma” package, followed by immune infiltration analysis using the CIBERSORT algorithm, led us to identify seven key genes. These genes underwent further scrutiny through consensus clustering and gene set variation analysis (GSVA). We developed a prognostic model using Lasso Cox regression and multivariable Cox analysis, which was then validated with a nomogram, predicting survival rates for lung adenocarcinoma. The model’s accuracy and universality were corroborated by ROC curves. Additionally, we explored the relationship between immune checkpoint genes and immune cell infiltration and identified two key genes, HLA-DQB1 and OLR1. This highlighted their potential as therapeutic targets. Our comprehensive approach sheds light on the molecular landscape of lung adenocarcinoma and offers insights into potential treatment strategies, emphasizing the importance of integrating single-cell and genomic data in cancer research.

## INTRODUCTION

Lung cancer, one of the most common and lethal malignancies worldwide, poses a significant threat to human health [1]. Smoking is considered the primary risk factor for lung cancer. Despite smoking being a major cause, other factors such as air pollution, exposure to asbestos, radon, nickel, arsenic, soot, and tar also significantly impact the incidence of lung cancer. Symptoms of lung cancer include dyspnea, chest discomfort, wheezing, bloody mucus, and hoarseness, along with more subtle symptoms like fatigue, loss of appetite, and unexplained weight loss [2]. Lung cancer is primarily categorized into two types: non-small cell lung cancer and small cell lung cancer. The distribution of pathological types varies in different regions; for instance, previous studies have shown a higher proportion of squamous carcinoma among patients who smoke and drink [3]. With advances in etiological research and precision medicine, the incidence and mortality rates of lung cancer are changing globally. Data from Canada indicate a significant reduction in lung cancer mortality rates by approximately 4% annually since 2015. Understanding the epidemiological characteristics of lung cancer is crucial for its prevention and treatment. Current treatments for lung cancer include minimally invasive surgery, targeted therapy, and immunotherapy [4]. Transcriptomics plays a vital role in the treatment of lung cancer by identifying genes closely associated with lung cancer through comparisons between the genotypes of lung cancer and normal lung tissues [5]. Recent studies also highlight the safety and effectiveness of personalized tumor treatments, such as the application of long non-coding RNA (lncRNA) in the treatment of lung cancer [6].

Autophagy, a self-repair and self-cleaning mechanism in cells, and single-cell research play a crucial role in lung cancer, offering insights into the complexity of the disease and the development of new treatment strategies. Autophagy demonstrates a complex “double-edged sword” effect in the progression and treatment of lung cancer [7]. It can suppress tumors by clearing damaged organelles, maintaining cellular homeostasis, and protecting normal cells. However, it also plays a role in the survival and drug resistance of tumor cells. For instance, EGFR-TKI drugs, well-studied in tumor-targeted therapy, can induce autophagy in tumor cells. The effect of autophagy here is twofold, sometimes promoting the survival of tumor cells [8]. Future research should focus on developing new models for studying autophagy, investigating the specific mechanisms of autophagy as a cell death or survival function in EGFR inhibition, and strategies for combining EGFR-TKI with autophagy-regulating drugs at different stages of the tumor and treatment [9]. Single-cell sequencing technology also plays a key role in lung cancer research, allowing researchers to reveal the molecular mechanisms of tumor cell metastasis and identify new circulating tumor cell (CTC) biomarkers [10]. For example, whole-genome sequencing has revealed cancer-associated single nucleotide variations. Moreover, bioinformatics analysis based on the TCGA database has successfully constructed a prognostic risk-scoring model for lung adenocarcinoma based on autophagy-related genes, providing new strategies for personalized treatment of lung cancer [11]. In summary, autophagy and single-cell research offer new perspectives and directions for the treatment of lung cancer, helping develop more effective treatment methods. Through these studies, we can gain a deeper understanding of the biological mechanisms of lung cancer, offering more precise treatment options for patients.

## METHODS

### Data collection and organization

We sourced our data from the Gene Expression Omnibus (GEO; https://www.ncbi.nlm.nih.gov/geo/) and The Cancer Genome Atlas (TCGA) (https://portal.gdc.cancer.gov/) databases [12]. Single-cell data for lung adenocarcinoma were obtained from the GEO database, specifically the GSE117570 dataset, for subsequent analysis. From TCGA, we included 31 normal samples and 538 lung cancer samples for further study. A total of 3076 autophagy-related genes were identified from the GENECARD database, along with the corresponding clinical pathological information downloaded from TCGA [13].

### Single-cell data analysis

The data from the GEO database were processed and analyzed for single-cell data using Seurat, dplyr, tidyverse, and data tables were employed for data handling and transformation; and ggplot2, ggpubr, and ggsci were used for data visualization [14]. To assess data quality, we calculated the expression ratio of mitochondrial genes and ribosomal protein genes in each cell. Based on these ratios, potential low-quality cells were filtered out. Subsequently, data from all samples were merged into a single Seurat object for unified downstream analysis. We then normalized the data, selected variable features, and conducted Principal Component Analysis (PCA) [15]. PCA helped us understand the main sources of variation in the data and provided a basis for subsequent clustering analysis. We also performed non-linear dimensionality reduction using the t-SNE algorithm for better visualization of cell similarities. Finally, cells were clustered based on expression characteristics, and different cell populations were identified and annotated using specific marker genes. Key genes in monocyte cells were selected for further analysis [16].

### Key gene selection

Differential analysis of TCGA data was performed using the “Limma” package in R [17], followed by an immune infiltration analysis. We then estimated the proportion of immune cell infiltration in each lung cancer sample using the CIBERSORT algorithm [18]. Based on the results of immune infiltration, a weighted gene co-expression network analysis was performed using the `WGCNA` package [19]. We first conducted sample clustering to detect and remove outliers and then chose an appropriate soft threshold to ensure a scale-free network distribution. Modules were constructed, and those related to monocyte cells were identified. Intersection analysis was then conducted among autophagy genes, WGCNA genes, and monocyte cell-related genes from single-cell data.

### Consensus clustering analysis of key genes

From the data obtained above, 7 key genes were identified for further analysis. All these genes were present in TCGA. We performed a consistent unsupervised clustering analysis using the “ConsensusClusterPlus” package in R [20], employing the K-Means algorithm to generate different clustering subtypes based on the expression of key genes. Each cluster had a significant sample size. The clusters exhibited high intra-cluster similarity and low inter-cluster similarity. To uncover the biological differences between molecular subtypes, gene set variation analysis (GSVA) was performed using gene sets compiled from MSigDB (C2.Cp.ke.v7.2) [21].

### Comprehensive analysis of different molecular subtypes

To evaluate the differences between the two clusters, we explored the association of different clusters with the prognosis and clinical characteristics of lung adenocarcinoma patients. Kaplan-Meier curves generated using the R packages “survival” and “survminer” were used to compare survival times [22].

### Model construction

Next, we constructed a prognostic model using these 7 key genes. The “glmnet” package in R was used for Lasso Cox regression analysis to reduce the risk of overfitting related to prognostic genes. Multivariable Cox analysis was employed to select candidate model genes and develop a prognostic model, which was validated based on the training set [23]. The Key Gene-Score was calculated as follows: Σ (Coefi × Expi), where Coefi represents the risk coefficient, and Expi represents the expression of each gene. Patients were divided into two groups based on the median risk score for Kaplan-Meier survival analysis and receiver operating characteristic (ROC) curve analysis. The “ggplot2” package was used for PCA (Principal Component Analysis) and t-SNE (t-distributed Stochastic Neighbor Embedding) analysis [24]. Subsequently, the dataset was divided into high-risk and low-risk groups, and KM survival analysis and ROC curves for each group were performed to validate the universality and accuracy of the model [25].

### Development of a nomogram

A nomogram was created using the “nomoR” package, incorporating the risk score and clinical characteristics of lung adenocarcinoma patients [26]. Each variable was assigned a score, and the total score for each sample was calculated by summing these scores. Calibration plots were used to depict the predicted values of 1-year, 3-year, and 5-year survival events compared to the virtual observed values. Finally, gene immune checkpoint analysis was conducted to distinguish between the high and low groups, followed by a drug sensitivity analysis.

## RESULTS

### Differential analysis and WGCNA analysis

**Figure 1:**
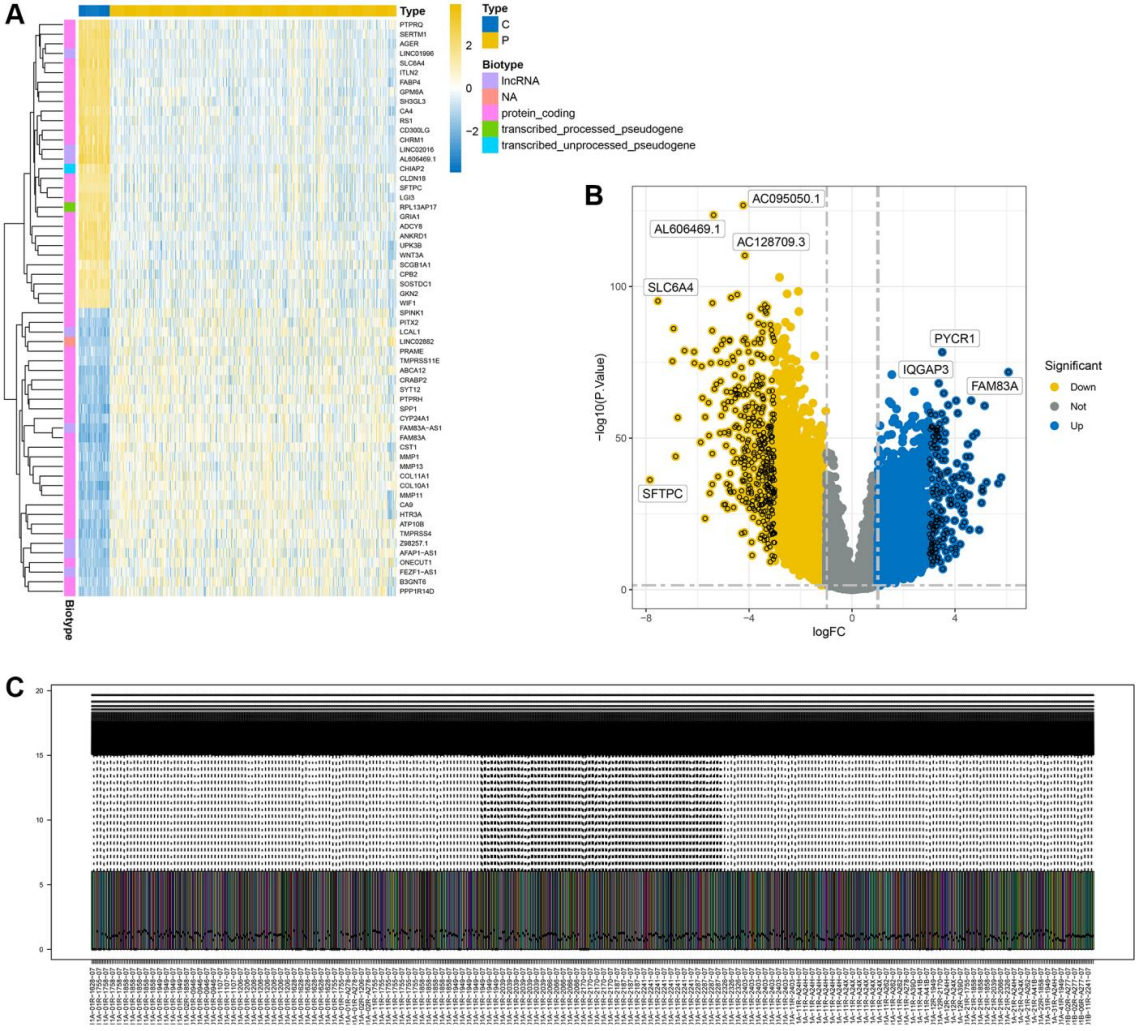
**Display of differential analysis results.** (**A**) Heatmap of differential genes; (**B**) Volcano plot of differential genes; (**C**) Distribution of differential genes after normalization.

We conducted a differential analysis of tumor data from The Cancer Genome Atlas (TCGA), categorizing adjacent tissue as the normal group and the cancer tissue as the diseased group. Using a threshold of *p* < 0.05 and an absolute logfc value greater than 1, we identified 7400 differential genes. Figure 1A displays a heatmap of these differential genes, while Figure 1B shows a volcano plot of the same. Figure 1C presents the results of data quality control post-normalization.

**Figure 2:**
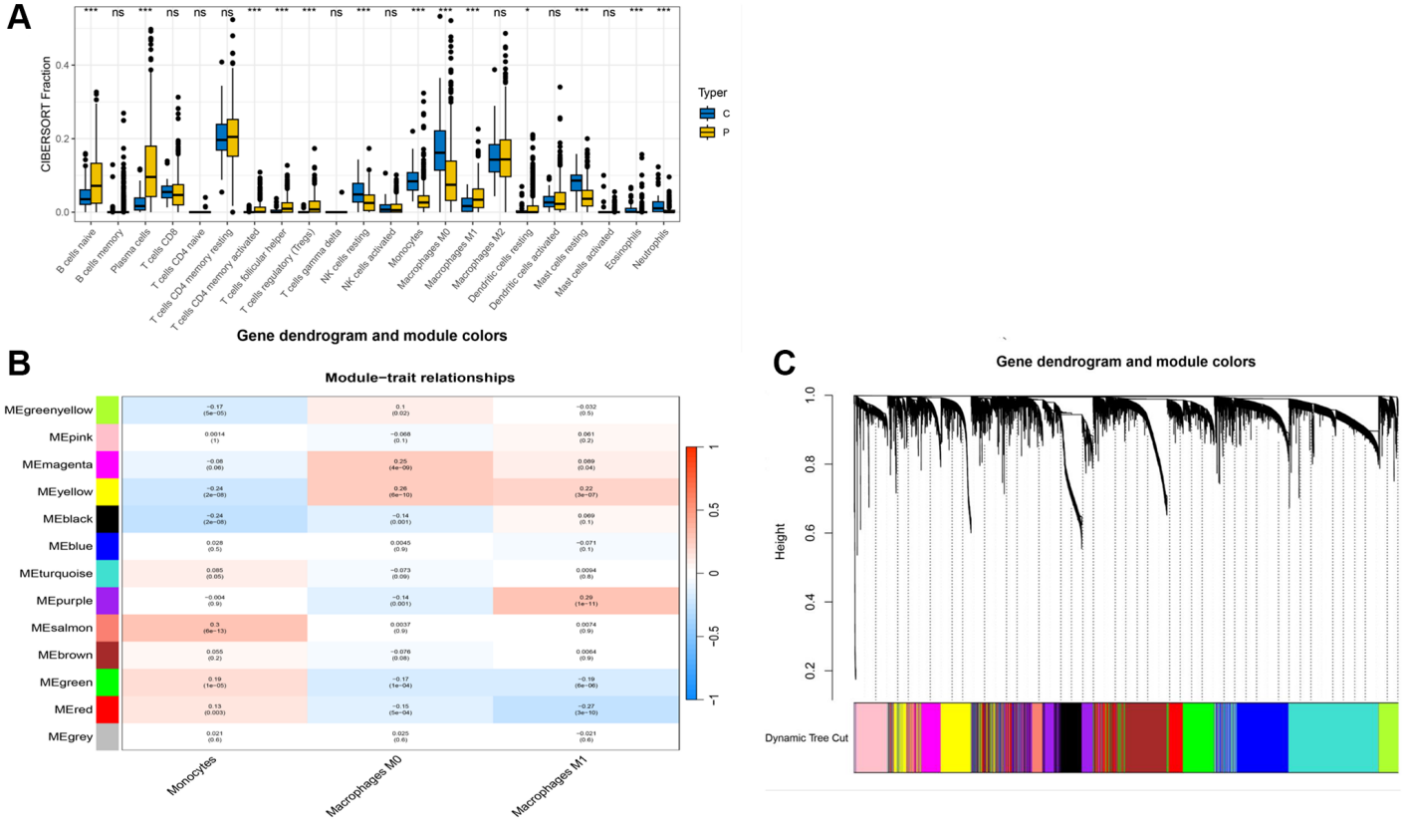
**WGCNA analysis results.** (**A**) Box plot of immune cell differences between groups; (**B**) Selection of modules associated with immune cells; (**C**) Distribution of subgroups in data.

Further, we conducted an immune infiltration analysis of the TCGA data. A differential analysis of immune cells between the normal and diseased groups is displayed in Figure 2A. Significant differences were observed in various cell clusters, such as naïve B cells, plasma cells, activated memory CD4 T cells, M0 macrophages, and notably, monocytes. We chose to focus primarily on monocytes for further analysis. Using the WGCNA method, we analyzed the monocyte cell-related modules in the immune process (Figure 2B), dividing cells into different modules based on monocyte cell scoring. Genes from modules with a *p*-value less than 0.05 were selected for subsequent analysis. Figure 2C shows the results of clustering.

### Key gene selection

**Figure 3:**
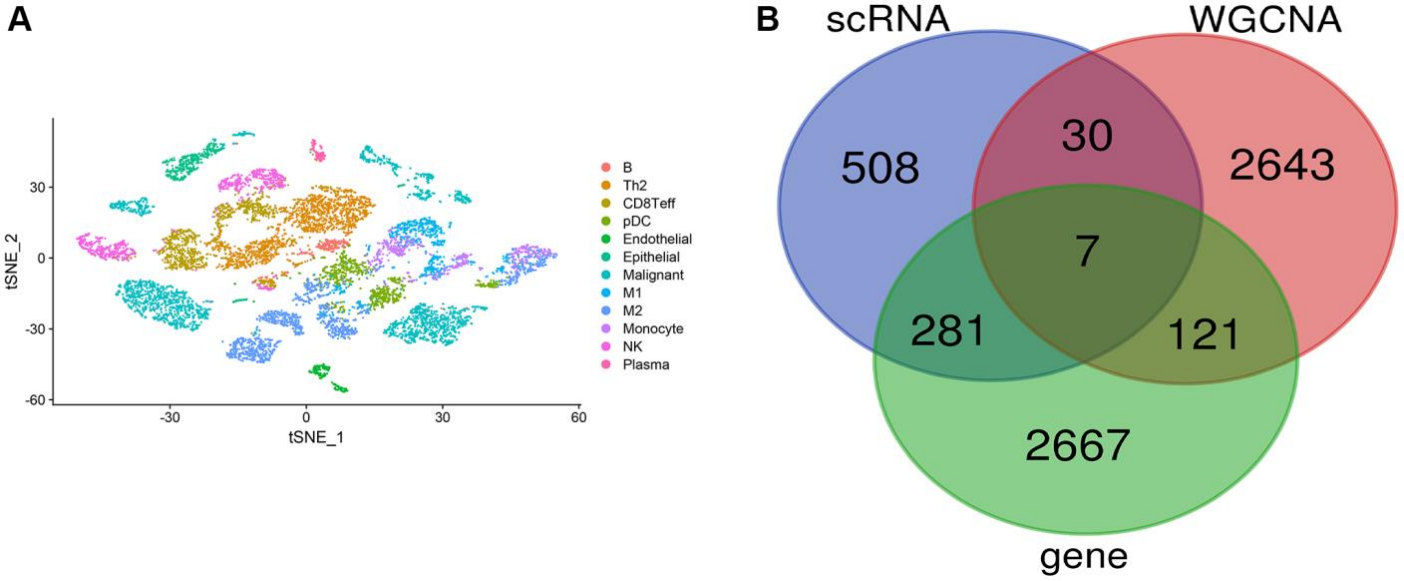
**Single-cell data analysis.** (**A**) Clustering annotation results of single-cell data; (**B**) Venn diagram for key gene selection.

We downloaded single-cell data from GSE117570, with the clustering annotation results shown in Figure 3A, dividing cells into 10 distinct clusters, including M1, M2, and monocyte cells. We selected genes from monocyte cells for further analysis. Next, we intersected genes from single-cell monocyte cells, genes identified through WGCNA, and autophagy-related genes, as illustrated in Figure 3B, yielding 7 key genes: OLR1, TREM1, SERPINA1, HLA-DRB5, HLA-DQA2, LYZ, and HLA-DQB1.

### Identification of key gene-related subtypes in lung cancer

**Figure 4:**
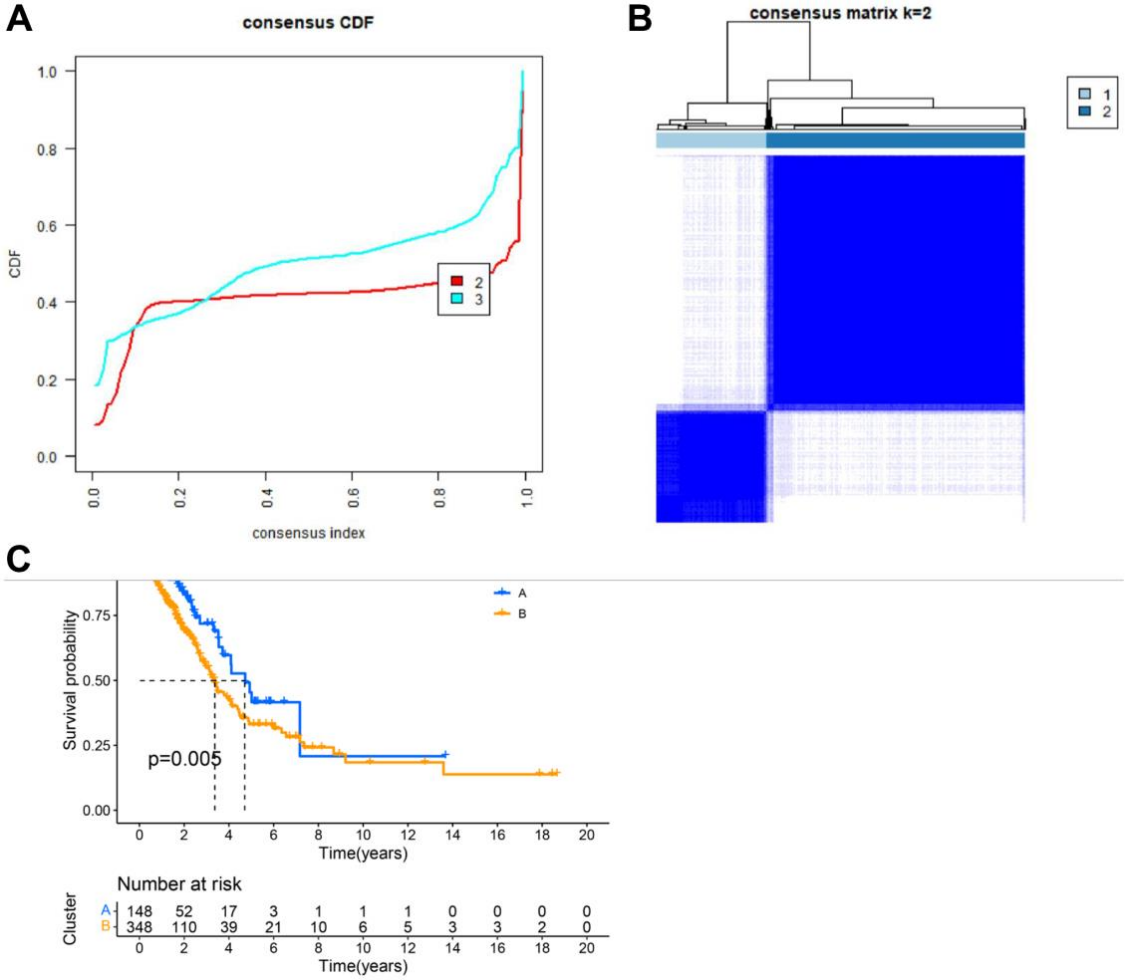
**Clustering analysis results.** (**A**) CDF plot of clustering; (**B**) Display of clustering results. (**C**) Survival analysis of different subgroups.

Having identified 7 key genes from the lung cancer dataset, we assessed their prognostic value using univariate Cox regression and Kaplan-Meier analysis, selecting genes with a *P*-value < 0.05. We then classified lung cancer patients based on the expression profiles of these 7 key genes using a consensus clustering algorithm (Figure 4A, 4B). Our findings indicated that k = 2 was the optimal variable for dividing the dataset into clusters A and B (Figure 4B). Survival analysis of these two clusters indicated a significant difference, as shown in Figure 4C.

**Figure 5:**
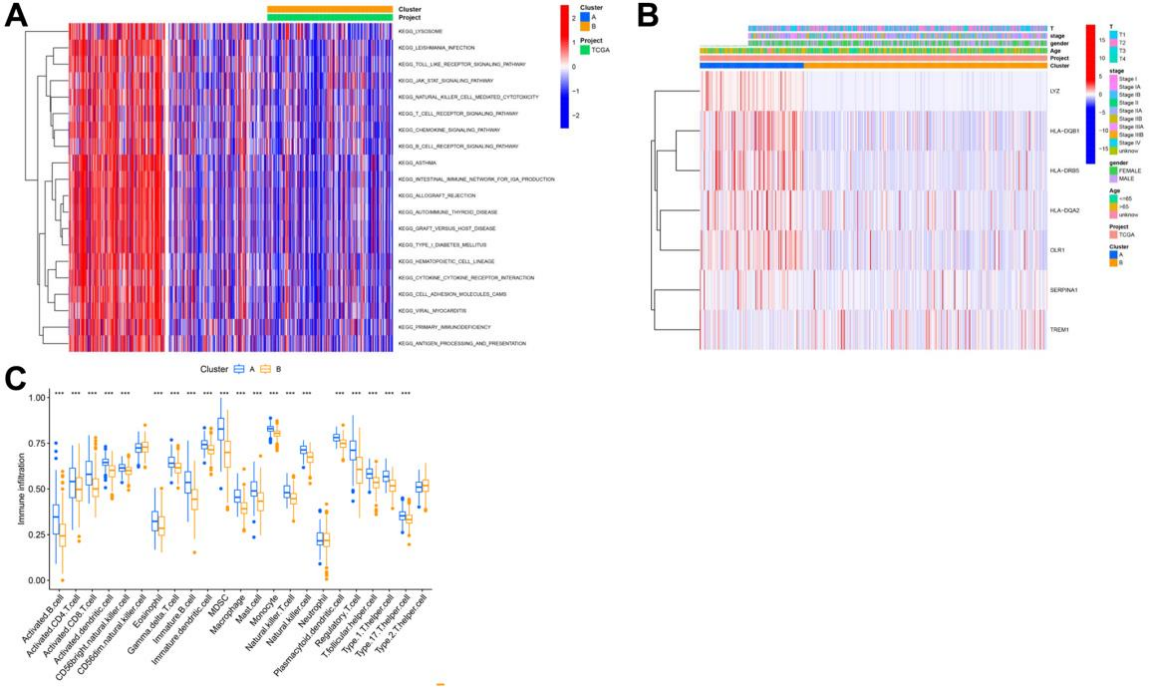
**Analysis of subtypes based on key genes.** (**A**) GSEA analysis results of different cluster outcomes. (**B**) Heatmap of cluster subgroups and clinical characteristics; (**C**) ssgsea analysis results of different clusters.

Further, we conducted a GSVA analysis of these two clusters. It was observed that these clusters exhibited significant differences in pathways such as KEGG_LEISHMANIA_INFECTION, KEGG_JAK_STAT_SIGNALING_PATHWAY, KEGG_T_CELL_RECEPTOR_SIGNALING_PATHWAY, and KEGG_INTESTINAL_IMMUNE_NETWORK_FOR_IGA_PRODUCTION (Figure 5A). The heatmap in Figure 5B shows the correlation of these clusters with clinical information of lung cancer patients. Additionally, an ssgsea analysis revealed notable differences between these clusters in various immune cells, such as Activated CD4 T cell, Activated CD8 T cell, Monocyte, etc., (Figure 5C).

### Model analysis

**Figure 6:**
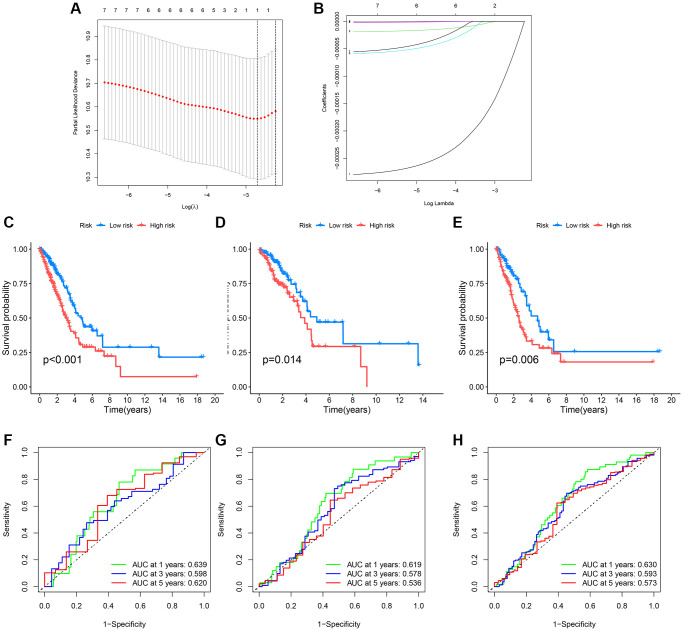
**Survival curves and ROC analysis of the risk model in training and testing groups.** (**A**, **B**) Lasso analysis display; (**C**–**E**) Survival curves showing prognostic survival status for different models; (**F**–**H**) ROC curves predicting 1-year, 3-year, and 5-year survival sensitivity and specificity based on risk scores in training, testing, and entire cohorts.

Initially, lung cancer patients were randomly divided into a training set and a test set in a 7:3 ratio. The best model was then selected through LASSO regression analysis, leaving the minimal likelihood deviation of genes and multivariable Cox regression analysis based on the Akaike information criterion (AIC) value (Figure 6A, 6B). Patients with a CRG_score lower than the median risk score were considered low-risk, while those with scores above the median were considered high-risk. To assess the model’s predictive capability, we calculated scores for the test set and the entire cohort and conducted ROC analysis to verify predictive accuracy. ROC curves in Figure 6F–6H show that the CRG-Score’s 1-year, 3-year, and 5-year survival rates in the training cohort were 0.630, 0.593, and 0.573, respectively. In the validation cohort, the 1-year, 3-year, and 5-year survival rates were 0.639, 0.598, and 0.620, respectively, with survival curves of all three groups indicating significance (Figure 6C–6E).

**Figure 7:**
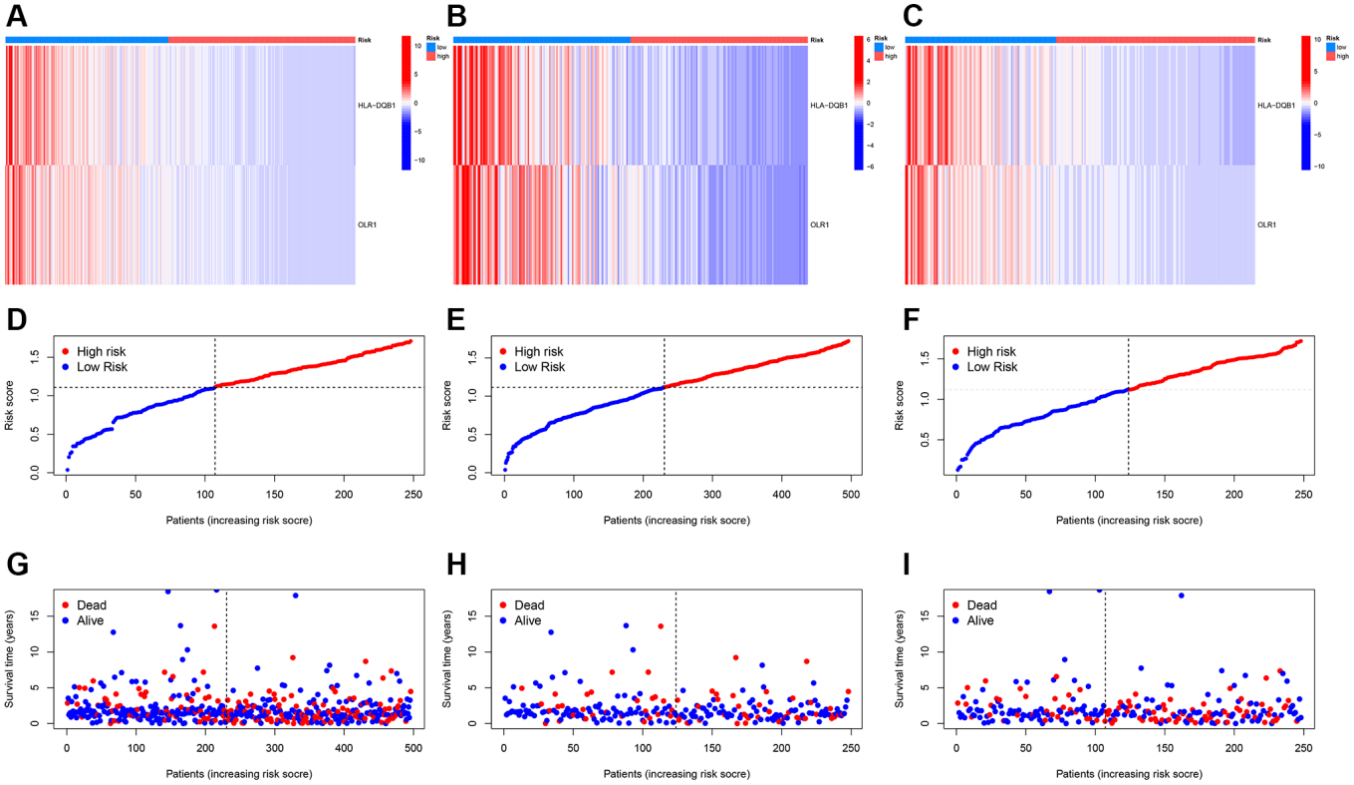
**Risk heatmap.** (**A**–**C**) Risk heatmap display for different groups; (**D**–**I**) Survival time and survival status between low-risk and high-risk groups in training, testing, and entire cohorts.

Further, we created heatmaps, PCA, and t-SNE distribution diagrams for the key genes, demonstrating significant dimensions of high and low score ancestors. Figure 7A–7C show heatmaps of key genes, while Figure 7D–7I display survival status and survival time between different models.

### Nomogram and immune analysis

**Figure 8:**
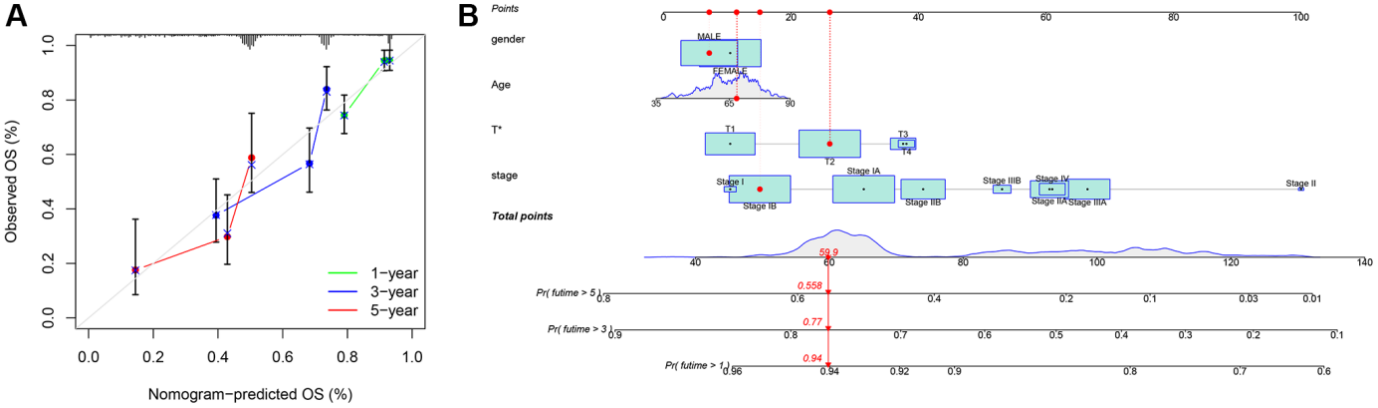
**Nomogram.** (**A**) Nomogram calibration chart; (**B**) Model nomogram.

For clinical application, we created a nomogram to estimate the survival rate of lung cancer patients, based on the correlation between risk scores and patient prognosis. Using this nomogram, we estimated the 1-year, 3-year, and 5-year OS (Figure 8A). Furthermore, we compared the predictive accuracy of the nomogram with other clinical variables, indicating that the nomogram provided better survival prediction (Figure 8B).

**Figure 9:**
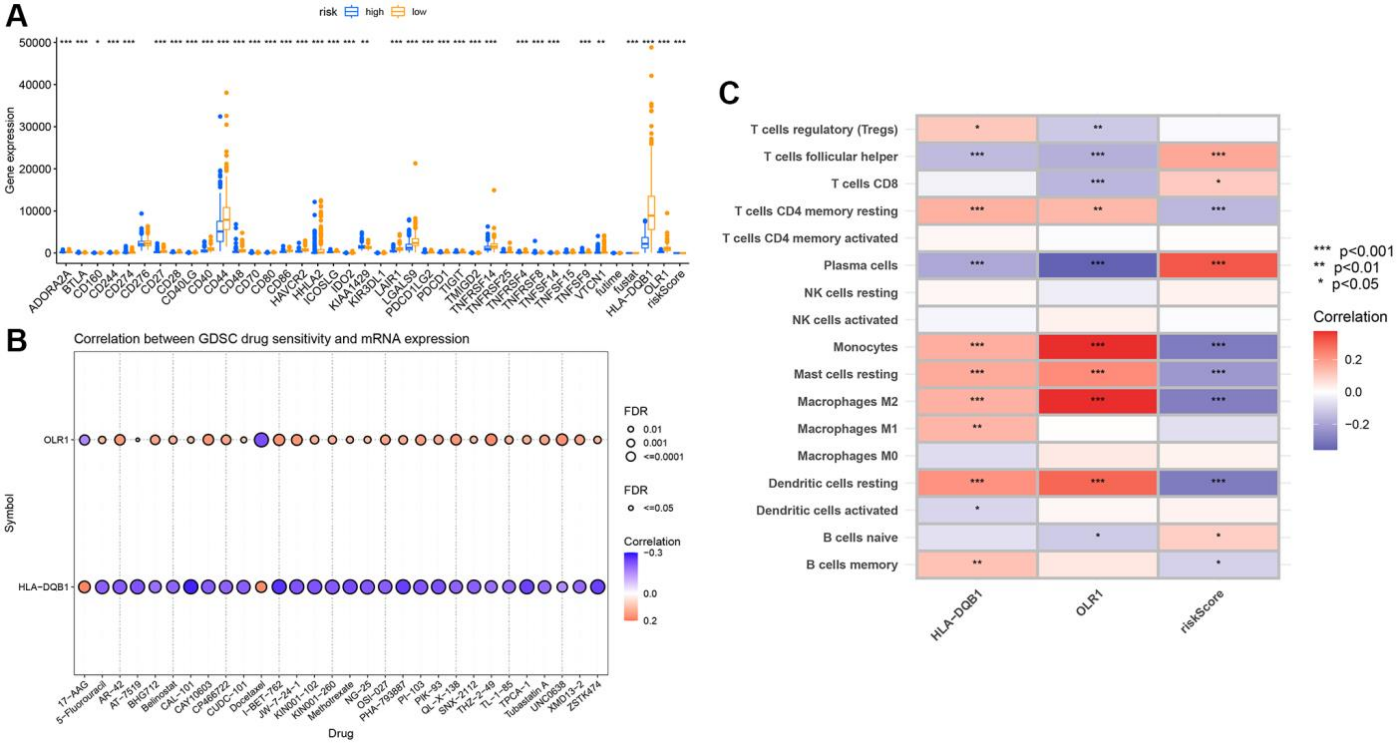
**Immune infiltration analysis of key genes.** (**A**) Immune checkpoint differential box plot; (**B**) Correlation analysis between key genes and immune infiltration; (**C**) Drug sensitivity analysis.

We first examined the expression relationship of immune checkpoint genes between high and low groups, noting significant differences in several immune checkpoints (Figure 9A). Using CIBERSORT, we associated key genes with immune cell infiltration. Stars indicate significant correlations; the deeper the color, the stronger the correlation, as evident with these two genes showing clear relevance to monocytes (Figure 9B), and most being significantly correlated with immune cells. Drug sensitivity analysis revealed these key genes to have apparent correlations with several drugs (Figure 9C).

## DISCUSSION

In lung cancer research, monocytes, particularly inflammatory monocytes, play a crucial role in the progression of the disease. Studies have linked these cells with lung squamous carcinoma, where they contribute to tumor growth through the promotion of fibrin cross-linking [27]. These monocytes are intimately associated with secretory-type lung cancers, influencing the tumor microenvironment by fostering tumor growth, angiogenesis, metastasis, chemotherapy resistance, and immune suppression. Moreover, alterations in these cells within the tumor microenvironment are tied to overall patient survival, especially as they differentiate into tumor-associated macrophages and dendritic cells. The current limitations in lung cancer treatment include an insufficient understanding of the disease’s complex heterogeneity and resistance to existing therapies. The development of precision target therapies, especially the introduction of immune checkpoint inhibitors, has revolutionized lung cancer treatment [28]. However, the success rate remains limited, with up to 70% of patients being non-responsive to these treatments. Thus, monocytes and their derivatives, such as tumor-associated macrophages, emerge as potential new therapeutic targets. A deeper understanding of the role of monocytes in the tumor microenvironment could lead to more effective treatment strategies, thereby improving the prognosis for lung cancer patients [29].

In a recent study on lung cancer, we made significant discoveries through an integrated analysis of data from the Gene Expression Omnibus (GEO) and The Cancer Genome Atlas (TCGA). Initially, we harnessed single-cell data for lung adenocarcinoma from the GEO database, particularly the GSE117570 dataset, along with lung cancer sample data from TCGA, which provided a wealth of information for our investigation. Delving deep into this data, we identified 3076 autophagy-related genes and extracted relevant clinical pathological information. In terms of single-cell data analysis, we employed tools such as Seurat for data quality control, normalization, and Principal Component Analysis (PCA), as well as t-SNE for non-linear dimension reduction. These steps enabled us to clearly identify and annotate different cell populations, especially monocytes, laying a crucial foundation for our subsequent research. Moving forward, we used the “Limma” package for differential analysis of TCGA data and coupled it with the CIBERSORT algorithm to estimate the proportion of immune cell infiltration in lung cancer samples. These analyses highlighted the significant role of monocytes in lung cancer. Through weighted gene co-expression network analysis using the WGCNA package, we further identified gene modules closely related to monocytes and delved into these genes.

Key findings include the selection of 7 critical genes through consensus clustering analysis, upon which we built a prognostic model for lung cancer. The Lasso Cox regression and multivariable Cox analysis helped reduce the risk of overfitting in our model, ensuring its accuracy and reliability. Additionally, we developed a predictive nomogram and validated its accuracy in forecasting 1-year, 3-year, and 5-year survival events using calibration plots. Among the key genes identified were HLA-DQB1 and OLR1. The HLA-DQB1 gene, a part of the human leukocyte antigen (HLA) complex gene family, encodes a vital protein in the immune system, crucial for distinguishing self from non-self proteins. Studies have shown that lower expression levels of HLA-DQB1 in lung adenocarcinoma tissue correlate with a reduced recurrence rate in patients, indicating its significant role in the immune response and prognosis of lung adenocarcinoma [30]. The OLR1 gene, coding for an oxidized low-density lipoprotein receptor, is associated with tumor metastasis and apoptosis. Although its direct link to lung cancer is not explicitly established, its role in tumor biology suggests a potential impact on lung cancer progression. These two genes could play key roles in the pathogenesis and treatment of lung cancer [31]. However, our study has limitations. Since the data were sourced from public databases, we couldn’t control the quality and consistency of sample collection and processing. Moreover, our research focused mainly on gene expression data, while other aspects of the lung cancer microenvironment, such as intercellular communication and the role of the tumor extracellular matrix, are also critical factors affecting treatment outcomes. Future research will require validation in larger, independent sample cohorts and should consider these additional aspects of the tumor microenvironment.
